# Translation and validation of the Persian version of the perception to care in acute situations (PCAS-P) scale in novice nurses

**DOI:** 10.1186/s12912-024-01760-z

**Published:** 2024-02-08

**Authors:** Reza Nemati-Vakilabad, Maryam Khoshbakht-Pishkhani, Saman Maroufizadeh, Nazila Javadi-Pashaki

**Affiliations:** 1 Student Research Committee, School of Nursing and Midwifery, Guilan University of Medical Sciences, Rasht, Iran; 2 Department of Medical-Surgical Nursing, School of Nursing and Midwifery, Guilan University of Medical Sciences, Rasht, Iran; 3 Department of Biostatistics and Epidemiology, School of Health, Guilan University of Medical Sciences, Rasht, Iran; 4 Social Determinants of Health Research Center (SDHRC), Guilan University of Medical Sciences, Rasht, Iran

**Keywords:** Nursing, Novice nurse, Educational measurement, Psychometrics, Acute care

## Abstract

**Background:**

Novice nurses providing care in acute conditions should have satisfactory performance. Accurate and appropriate evaluation of the performance of novice nurses in providing care in acute situations is essential for planning interventions to improve the quality of patient care. This study was conducted to translate and evaluate the psychometric properties of the Persian version of the Perception to Care in Acute Situations (PCAS-P) scale in novice nurses.

**Methods:**

In this methodological study, 236 novice nurses were selected by the convenience sampling method. 17-item scale PCAS-P was translated into Persian by the forward-backward process. Then, this version was used for psychometric evaluation. For this purpose, face validity, content validity, and construct validity were assessed using confirmatory factor analysis. Internal consistency and stability reliability were calculated. The data were analyzed using SPSS and AMOS software.

**Results:**

The PCAS-P scale maintained the meaning of the original English version and was clear, explicit, and understandable for novice nurses. Confirmatory factor analysis showed that this Persian version is consistent with the proposed model and confirmed the fit of the three-factor model. The values of Cronbach’s alpha coefficient, McDonald’s omega, Coefficient *H*, and average inter-item correlation were excellent for the overall scale and its dimensions, and the three latent factors had good convergent and discriminant validity. Additionally, the average measurement size was 0.944 ICC (95% CI 0.909 to 0.969).

**Conclusion:**

The PCAS-P scale is valid and reliable for measuring novice nurses’ perception of acute situations.

**Supplementary Information:**

The online version contains supplementary material available at 10.1186/s12912-024-01760-z.

## Introduction

Nurses are at the forefront of diagnosing and managing conditions that are potentially life-threatening to patients [1]. Therefore, they should identify the conditions that indicate the severity of the deterioration of the patients and respond to them effectively and quickly [2]. One of the areas where nurses play a crucial role is in acute situations [3]. The term “acute situations” generally describes a wide range of urgent and critical events that require immediate attention and intervention to prevent severe harm or consequences [4]. Nursing care in acute situations requires high-level skills and abilities from nurses due to the instability and unpredictability of the problem, which poses a challenge for novice nurses and holds greater importance [5].

As per the definition provided by the American Nurses Association (ANA), novice nurses are those individuals who have just entered the nursing profession, have less than a year of clinical experience, and are in the early stages of developing their professional skills and knowledge [4]. In the “From Novice to Expert” model, Benner describes experience as something beyond the passage of time. This model assumes that nurses pass through five skill levels, from relying on abstract principles to applying tangible experiences [6]. Benner claims that most newly graduated nurses, novice or, at best, advanced beginners, can hardly provide acceptable care. He suggests that gaining experience in different conditions is the main factor in developing and improving their expertise [7]. Despite receiving comprehensive theoretical training during their studies, nurses should be fully prepared to enter clinical environments after graduation [8]. One of the problems they experience is a feeling of unpreparedness to work in a clinical setting [3].

Insufficient readiness and timely response to acute situations can have irreparable consequences [9]. This issue becomes more complicated with the increase in patients’ age and care needs because the quality of care is also expected to increase [10]. An integrative review study showed that insufficient readiness increases errors impacting patient safety [11]. Additionally, according to Hawkins et al., novice nurses transitioning to acute care settings are influenced by fear, such as fear of making mistakes, harming patients, and not meeting expectations [12]. This overwhelming feeling may be due to their limited clinical experience and inability to focus on what matters in acute situations [6].

Furthermore, disregarding novice nurses’ ability to manage acute situations can affect the quality of patient care and lead to psychological stress, job dissatisfaction, and turnover among novice nurses [13]. Given that novice nurses have entered a new phase in their profession, they often experience transition shock, fear, and anxiety, which can lead to errors and serious consequences [14]. Identifying weaknesses allows implementing interventions to improve novice nurses’ skills, confidence, and clinical competency in acute situations [15]. Consequently, having a valid tool to assess novice nurses’ perception of caring in acute situations is essential for planning based on their capabilities [16].

Due to the importance of novice nurses’ perception of providing acute care, various self-report tools assess nurses’ competence and ability in various nursing fields but do not precisely measure their perception of acute care. Despite this, nurses’ competency tools were often developed and designed based on national goals and guidelines [17, 18] or theoretical frameworks [19], but they mostly have limitations. For example, these tools lack items about safety and evidence-based performance in various clinical situations, which are core competencies in the nursing profession. Furthermore, some of these tools have a fixed (not randomized) order of items, which may cause a proximity effect and limit the generalizability of results in different clinical situations, including acute situations. As a result, to address these limitations, in 2020, a tool called the perception to care in acute situations (PCAS) in novice nurses was designed by Sterner et al. [16]. The tool items were developed using an inductive approach based on previous qualitative studies [1, 3] that involved novice nurses as participants.

However, according to the authors’ knowledge, there is currently no measurement scale to assess the perceptions of novice nurses regarding their ability to provide care in situations described as acute among the population of Iranian novice nurses. Such a scale would facilitate the evaluation of educational interventions and guide novice nurses to reflect on areas they feel are problematic. This study was conducted to determine the psychometric properties of the Persian version of perception to care in acute situations (PCAS-P) Scale in novice nurses.

## Methods

### Design and setting

This methodology study examined the psychometric properties of the Persian version of the perception to care in acute situations (PCAS-P) Scale in novice nurses. The study was conducted using a convenience sampling method with novice nurses working in six educational-therapeutic hospitals in northern Iran from July to October 2023.

### Participants

Data were collected from 236 novice nurses in affiliated educational-therapeutic hospitals of Guilan University of Medical Sciences, who were included in the study using a convenience sampling method. The inclusion criteria for the study included the willingness to participate (voluntary consent), having a minimum bachelor’s degree in nursing, having less than one year of work experience, and having no history of neurological and psychiatric diseases (self-reported). The exclusion criteria for the study included incomplete questionnaire responses.

### Instrument

The first section of the tool consisted of a demographic information form, including age, working experience, gender, marital status, education level, working department, and type of university. The second section of the tool was the perception of care in acute situations (PCAS) scale among novice nurses, designed by Sterner et al. [16]. PCAS is a self-report tool consisting of 17 items and three subscales: confidence in the provision of care (10 items), communication (4 items), and patient perspective (3 items). The items are rated on a 4-point Likert scale. For items 1 to 3 as 1 = strongly disagree, 2 = somewhat agree, 3 = agree, 4 = strongly agree and items 4 to 17 as 1 = very poor, 2 = poor, 3 = good, 4 = very good. Higher scores indicate a greater perception of ability in acute care situations.

### Psychometric evaluation

#### Translation procedure

After emailing the scale developer (Dr. Anders Sterner), the researchers obtained permission to use PCAS among Iranian novice nurses. The scale was translated into Persian based on the guidelines of the World Health Organization (WHO) [20] and using the forward-backward method. In the first stage, the original version of the scale was translated into Persian by two professional translators familiar with nursing concepts and compared to the English version by research team members. After the initial translation, a standardized version was created. In the subsequent stage, a professional translator familiar with nursing concepts but unaware of the original tool translated the Persian version into English. The translated version was then sent to the developer for review to ensure that the main concepts, words, and meanings were accurately conveyed. After receiving and incorporating feedback from the tool developer, the final Persian version of the PCAS scale was prepared and evaluated for validity and reliability.

#### Face validity

We used qualitative and quantitative methods to evaluate the scale’s face validity. For the qualitative approach, we gave the PCAS scale to 10 novice nurses using purposive sampling. Qualitative face-to-face interviews were conducted to gather opinions on the items’ relevance, difficulty, and ambiguity. Based on their feedback, we made revisions to clarify problematic expressions.

For quantitative evaluation, the novice nurses evaluated the degree of appropriateness of each item based on a 5-point Likert scale: quite important = 5, important = 4, almost important = 3, a little important = 2, not important = 1. The researchers calculated the frequency of novice nurses giving scores of 4 or 5 and the average scores obtained from their responses to each item (Importance). Using this data, they calculated the impact score for each item by multiplying the frequency percentage with its importance score (***Impact score*** = Frequency (%) x Importance). An impact score ≥ 1.5 was considered appropriate for each item [21].

#### Content validity

We used qualitative and quantitative methods to evaluate the scale’s content validity. In the qualitative approach, we used purposive sampling to give the PCAS scale to 10 experts (4 nursing faculty members, three emergency medicine specialists, and three health in disasters and emergencies). After qualitative examination, they provided necessary feedback based on grammar, wording, item allocation, and scaling criteria.

Quantitative content validity was assessed by measuring the content validity ratio (CVR) and content validity index (CVI). For calculating CVR, the experts were asked to rate each item as either “essential”, “useful but not essential”, or “not essential” on a 3-point Likert scale. Afterwards, the Content Validity Ratio (CVR) was computed with the help of the following formula: ***CVR*** = (Ne– N/2)/(N/2), where N stands for the total number of experts invited to participate, and Ne denotes the number of experts who ranked the item. As per Lawshe’s table, a CVR value exceeding 0.62 is acceptable [22]. Additionally, for calculating CVI, experts were asked to determine each item’s relevance, clarity, and simplicity on a 4-point Likert scale: 1 = not relevant, 2 = somewhat relevant, 3 = relevant, and 4 = completely relevant. CVIs over 0.79 were considered acceptable [23].

To ensure the accuracy of the evaluation, we assessed the content validity by examining the floor and ceiling effects. When more than 15% of participants achieve the lowest or highest attainable score, there is a presence of floor or ceiling effect. Floor and ceiling effects exceeding 15% indicate that items representing the minimum or maximum intensity of the phenomenon are likely not included, which suggests insufficient content validity of the tool [24].

#### Construct validity

Since the PCAS scale is theory-based and developed using exploratory factor analysis (EFA), confirmatory factor analysis (CFA) was used in the current study to measure and determine the construct validity. CFA determines the fit between a hypothetical model and the data obtained from research samples [25]. Additionally, CFA indicates how well each item assesses the dimensions of the scale. Maximum likelihood estimation (MLE) was used to estimate the parameters. The evaluation of model fit indices was based on the following parameters: The ratio of chi-square to its degree of freedom (χ^2^/df) < 3, Root Mean Square Error of Approximation (RMSEA) < 0.08 [26], Goodness of Fit Index (GFI) > 0.90, Comparative Fit Index (CFI) > 0.90, Tucker-Lewis index (TLI) > 0.90, Incremental Fit Index (IFI) > 0.90, Adjusted Goodness of Fit Index (AGFI) > 0.80, and Parsimonious Normed Fit Index (PNFI) > 0.50 [27]. Notably, factor loadings above 0.3 and T-values above 1.96, which are statistically significant, were considered acceptable [28].

The literature has different opinions regarding the minimum required sample size for CFA. Some researchers have considered the required sample size based on the number of individuals. It has been suggested that the minimum sample size for conducting a CFA should be 100 participants [25] or even more than 100 participants [29]. Additionally, some researchers have recommended that the sample size range between 100 and 200 participants [30] or between 200 and 400 participants [31]. Finally, another study has suggested that the sample size should exceed 250 participants [32]. Some researchers argue that the minimum sample size will vary depending on the number of items in the measurement tool. According to one perspective, the minimum sample size should be approximately 3–6 times the total number of items in the instrument [33], while another view argues that it should be at least five times [28] or 50–100 participants per variable [34]. According to the abovementioned perspectives, the number of participants (sample size) and collected data in this study exceeded the minimum required sample size for CFA. Since there were 17 items in the PCAS scale, 15 participants were considered for each item. Overall, a convenience sampling method selected 255 novice nurses. After removing outliers and missing values, the response rate was 92.5%. Finally, valid data from 236 participants were analyzed, which seems sufficient and appropriate for CFA.

#### Convergent and discriminant validity

Calculating the convergent and discriminant validity of the PCAS scale was done using the Fornell and Larcker method as well as through Average Variance Extracted (AVE), Maximum Shared Squared Variance (MSV), and Composite Reliability (CR) [35]. When AVE values are high, the indices are suitable substitutes for the latent variable. To assess convergent validity, AVE values should be greater than 0.5, and CR values should be greater than 0.7 [36]. If AVE is less than 0.5, but CR is higher than 0.6, the intended construct still has sufficient convergent validity [35]. Additionally, AVE greater than MSV indicates good discriminant validity [28]. Hair et al. stated that convergent validity exists when the items of a construct are close to each other and have a high common variance. Furthermore, divergent validity exists when the items of a construct or extracted latent factors are entirely different and distant from each other [28].

#### Reliability

Internal consistency was assessed using data of construct validity. Cronbach’s alpha coefficient (α), McDonald’s omega (ω), Coefficient *H*, and Average Inter-Item Correlation (AIC) were calculated for the entire instrument and its dimensions. McDonald’s omega total (${{\omega }}_{t}$) is calculated through factor analysis using the following formula:

$${\omega _t} = \frac{{{{\left( {\sum {{\lambda _i}} } \right)}^2}}}{{{{\left( {\sum {{\lambda _i}} } \right)}^2} + \sum {{\theta _{ii}}} }}$$

Where $$ {\lambda }_{i}$$ is the factor loading (not necessarily standardized) for the $$ i$$th item on the scale, $$ {\theta }_{ii}$$ is the error variance for the $$ i$$th item, and $$ i$$ is the number of items from 1 to *k* [37]. A value of α, ω, and H greater than 0.7 was considered acceptable [38, 39]. We used Coefficient *H* to demonstrate the maximum reliability of subscales [37, 40]. When measuring internal consistency, AIC is independent of the number of items and sample size. We considered the average correlation between items acceptable within a minimum range of 0.30. A high inter-item correlation (> 0.80) indicates redundancy and is undesirable. Additionally, no meaningful structure is present if all correlations are close to zero [41].

The test-retest reliability method assessed stability based on the intraclass correlation coefficient (ICC) with a two-way random model. The questionnaire was calculated by collecting data from 30 novice nurses at a two-week interval, and ICC values of 0.75 or above were considered acceptable [42].

### Data analysis

Univariate distribution for outliers was examined through the assessment of skewness (± 3) and kurtosis (± 8), while multivariate outliers were examined through Mahalanobis squared distance (*p* < 0.001). The researchers assessed the normality of multivariate variables using Mardia’s coefficient, with a value greater than 8 indicating a departure from normal distribution [43].

The descriptive statistical analyses were conducted using IBM SPSS Statistics for Windows (version 26.0, IBM Corp., Armonk, NY, USA), and confirmatory factor analysis was performed using AMOS software (version 24.0, IBM Corp., Armonk, NY, USA). The significance level for statistical analysis was set at *p* < 0.05.

### Ethical considerations

The Ethics Committee of Guilan University of Medical Sciences approved the proposal of this study with the ethics code IR.GUMS.REC.1402.264. The translation process was carried out after obtaining written permission via email from the tool developer. The research project adhered to the principles outlined in the Declaration of Helsinki, ensuring participants were informed about the research’s objective, study method, nature, and duration before participating. We obtained written informed consent without any coercion or threats. Ethical considerations such as confidentiality, anonymity, and data privacy were also observed.

## Results

### Characteristics of participants

A total of 236 new nurses participated in this study. The participants’ mean (standard deviation) age was 25.63 ± 2.06 years. More than half of the participants were female (*n* = 125, 53%) and primarily single (*n* = 184, 78%). The demographic characteristics of the participants are summarized in Table 1.

**Table 1:** Demographic characteristics of the participants (*n* = 236)

Variable	Categories	Mean ± SD	
**Age** (year)		25.63 ± 2.02	
**Working experience** (month)		8.75 ± 2.36	
		**No.**	**Percentage**
**Gender**	Male	111	47.0
	Female	125	53.0
**Marital status**	Single	184	78.0
	Married	52	22.0
**Education level**	Bachelor’s degree	212	89.8
	Master’s degree	24	10.2
**Working department**	Medical	35	14.8
	Surgical	39	16.5
	Emergency	49	20.8
	ICU	45	19.1
	Pediatric	40	16.9
	Other	28	11.9
**Type of university**	State university	181	76.7
	Private university	55	23.3

### Translation phase

Each of the three professional experts independently re-evaluated the final translation of the PCAS-P scale with the original English version, being familiar with nursing concepts. The results showed that the PCAS-P scale preserved the original English version’s meaning, and the Persian version’s language was clear, explicit, and understandable.

### Face validity

Based on the feedback of novice nurses in the process of face validity, we made some minor revisions in terms of difficulty, relevancy, and ambiguity in the translated version of PCAS-P. As a result, all items obtained a score equal to or higher than 1.5 (ranging from 2.5 to 4.8) (Table 2). At this stage, all items were identified as necessary for novice nurses in the target group, and none were removed from the translated version of PCAS. Therefore, all items were retained for further stages.

**Table 2:** The results for the face and content validity of the PCAS-P (*n* = 236)

Item	Impact score	CVI			CVR
		Relevance	Clarity	Simplicity	
1	4.8	1	1	1	1
2	4.7	1	1	1	1
3	3.8	0.9	0.9	0.9	0.7
4	2.5	1	1	0.9	0.9
5	3.2	1	1	1	0.8
6	3.7	1	0.8	1	1
7	4.3	0.9	1	1	1
8	2.5	1	1	0.8	1
9	3.4	1	1	1	1
10	2.7	0.9	1	1	0.7
11	4.7	1	1	1	0.7
12	2.5	1	0.9	1	0.8
13	3.4	0.8	0.9	0.9	0.7
14	3.7	1	1	0.9	1
15	2.7	1	1	1	0.9
16	3.8	1	1	1	0.7
17	4.7	1	1	0.9	1

### Content validity

According to Lawshe’s table, all items had a CVR greater than 0.62. The CVI for all items using the Waltz and Bausell method was more significant than 0.79 (Table 2). In the qualitative phase, experts stated that the four-point response category had a suitable ranking scale. Other criteria, such as grammar, wording, item allocation, and scaling, were reported to be appropriate. Therefore, content validity was acceptable for each of the 17 items. There was no floor or ceiling effect (< 15%) for the total score and three subscales of PCAS-P (Table 3).

**Table 3:** Descriptive statistics, floor and ceiling effects of the 17-item PCAS-P (*n* = 236)

Dimensions	No. of item	Possible range	Mean ± SD	Skewness	Kurtosis	Floor effect (%)	Ceiling effect (%)
Confidence in provision of care	10	1–4	3.30 ± 0.52	− 1.17	-1.22	5 (2.1%)	15 (6.4%)
Communication	4	1–4	3.38 ± 0.53	− 1.32	− 1.38	2 (0.8%)	31 (13.1%)
Patient perspective	3	1–4	3.19 ± 0.60	− 1.46	− 1.59	20 (8.5%)	26 (11.0%)
Total	17	1–4	3.30 ± 0.48	-1.65	− 1. 74	1 (0.4%)	2 (0.8%)

### Descriptive statistics of the 17-item PCAS-P

Table 3 presents descriptive statistics for the Persian version of the 17-item Perception to Care in Acute Situations (PCAS-P) Scale. The overall mean score of the scale was 3.30 (0.48), while the mean scores for the subscales of confidence in the provision of care, communication, and patient perspective were 3.30 (0.52), 3.38 (0.53), and 3.19 (0.60) respectively. The results also indicated that the overall scale and subscales were negatively skewed, indicating positive perceptions among participants towards all items.

### Construct validity

**Fig. 1:**
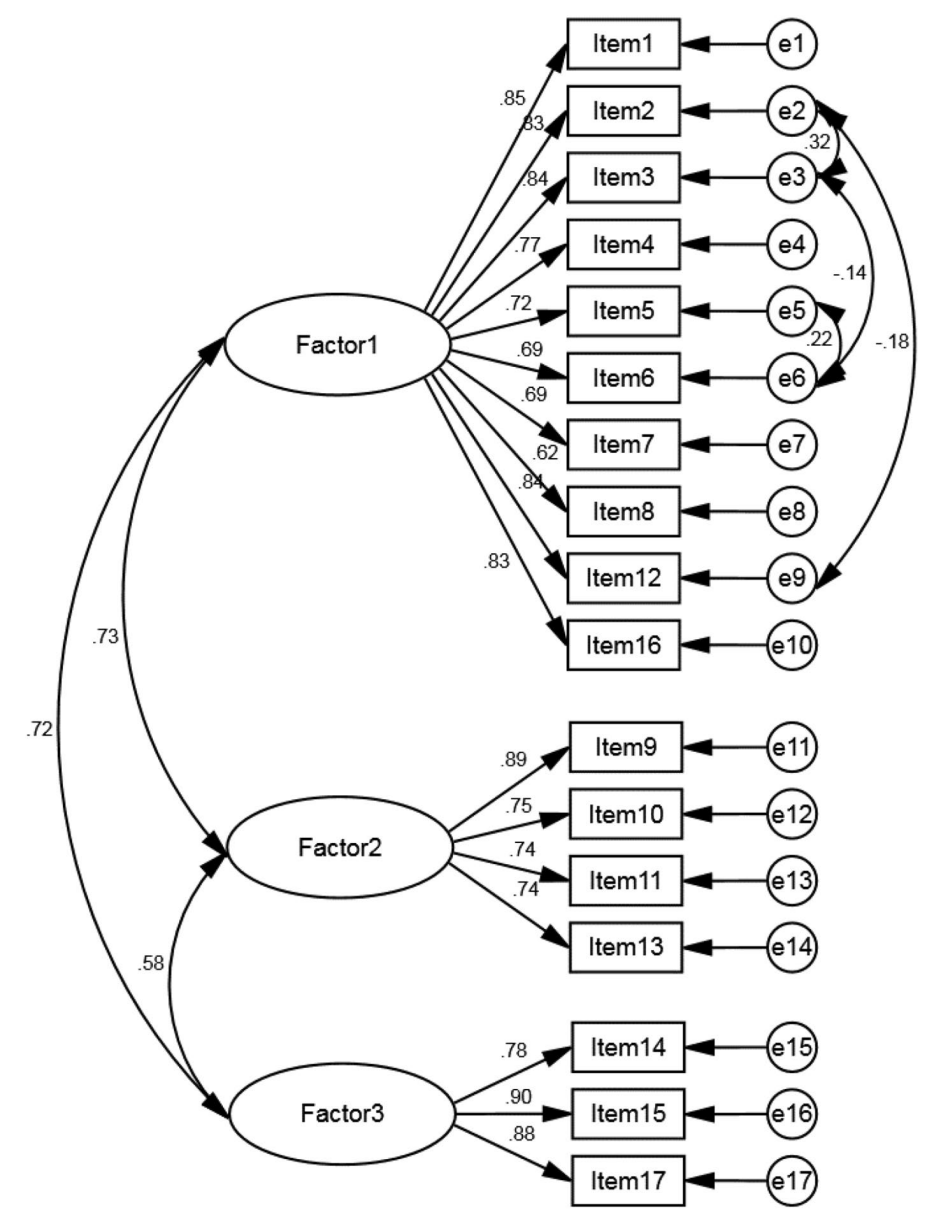
The confirmatory factor analysis model of the PCAS-P (*n* = 236)

To evaluate the construct validity of the tool, we utilized Confirmatory Factor Analysis (CFA). Figure 1 illustrates the structure of the Persian version of PCAS-P, where the latent factors 1 to 3 are confidence in the provision of care, communication, and patient perspective, respectively. We utilized covariance matrices and computed various goodness-of-fit indices. The CFA results for the three-factor model showed that all factor loadings for items were above 0.3 (ranging from 0.62 to 0.90), indicating that no items were removed (*p* < 0.001). Additionally, based on T-value tests, all relationships between latent factors and their corresponding items were significant (T-value > 1.96 for all items), and there was no heterogeneity, which means that all observed variables (items) were able to predict their respective latent factors. Based on goodness-of-fit indices, the proposed model and its constituent concepts are acceptable overall: χ^2^ = 213.27, df = 106, *p* < 0.001, RMSEA = 0.066, GFI = 0.902, CFI = 0.960, TLI = 0.952, IFI = 0.960, AGFI = 0.866, and PNFI = 0.761 (Table 4), and it is approved by three factors among novice nurses.

**Table 4:** Goodness of fit statistics for CFA models of the PCAS-P (*n* = 236)

Indices	χ^2^	df	*p*-value	χ^2^/df	RMSEA	GFI	CFI	TLI	IFI	AGFI	PNFI
CFA model	213.27	106	0.001	2.012	0.066	0.902	0.960	0.952	0.960	0.866	0.761
Acceptable values	-	-	> 0.05	< 3	< 0.08	> 0.90	> 0.90	> 0.90	> 0.90	> 0.80	> 0.50

Abbreviations: χ^2^/df, Ratio of chi-square to its degree of freedom; RMSEA, Root Mean Square Error of Approximation; GFI, Goodness of Fit Index; CFI, Comparative Fit Index; TLI, Tucker-Lewis index; IFI, Incremental Fit Index; AGFI, Adjusted Goodness of Fit Index; PNFI, Parsimonious Normed Fit Index

### Convergent and discriminant validity

Table 5 shows that CR and AVE values were greater than 0.7 and 0.5, respectively (CR > AVE), indicating good convergent validity for all factors. Additionally, the values of MSV for the latent factors were lower than AVE, confirming discriminant validity as well (Table 5).

**Table 5:** Indices of the convergent, discriminant validity, and reliability of the PCAS-P (*n* = 236)

Dimensions	CR	AVE	MSV	α	ω	H	AIC	ICC (95% CI)
Confidence in provision of care	0.936	0.596	0.533	0.936	0.939	0.727	0.592	0.941 (0.904 to 0.968)
Communication	0.863	0.612	0.533	0.860	0.864	0.752	0.604	0.869 (0.770 to 0.931)
Patient perspective	0.890	0.731	0.519	0.887	0.890	0.902	0.724	0.810 (0.651 to 0.903)
Total				0.947	0.948	0.971	0.512	0.944 (0.909 to 0.969)

Abbreviations: CR, Composite Reliability; AVE, Average Variance Extracted; MSV, Maximum Shared Squared Variance; α, Cronbach’s alpha; ω, McDonald’s omega coefficient; H, Coefficient *H*; AIC, Average Inter-Item Correlation; ICC, Intraclass correlation coefficient

### Reliability

The 17-item structure of the PCAS-P scale demonstrated excellent internal consistency (α = 0.947, ω = 0.948, coefficient *H* = 0.971, and AIC = 0.512). Ten items from factor 1 (α = 0.936, ω = 0.939, coefficient *H* = 0.727, and AIC = 0.592), as well as four items from factor 2 (α = 0.860, ω = 0.864, coefficient *H* = 0.752, and AIC = 0.604), and three items from factor 3 (α = 0.887, ω = 0.890, coefficient *H* = 0.902, and AIC = 0.724) also demonstrated excellent internal consistency (Table 5).

Test-retest was used to evaluate the stability of the instrument. The stability of the overall tool was (ICC = 0.944), confidence in the provision of care (ICC = 0.941), communication (ICC = 0.869), and patient perspective (ICC = 0.810) (Table 5).

### Production of the final model

After evaluating validity and reliability, the Persian version of PCAS was finalized with 17 items categorized into three dimensions. These dimensions included “confidence in the provision of care” with ten items, “communication” with four items, and “patient perspective” with three items.

## Discussion

This study aimed to translate and determine the psychometric properties of the Persian version of the Perception to Care in Acute Situations (PCAS) scale among novice nurses in affiliated educational-therapeutic hospitals of Guilan University of Medical Sciences, with the participation of 236 novice nurses. Such a scale can be used as a valuable tool to assess the relative effects of nursing education quality on better preparedness of novice nurses for acute situations, addressing the need expressed in previous studies that have called for new educational initiatives to better prepare novice nurses for clinical environments [44, 45].

The PCAS scale consists of 17 items that are categorized into three subscales. The three subscales of this scale are “confidence in the provision of care” (10 items), “communication” (4 items), and “patient perspective” (3 items), which are related to the provision of care in acute situations. Najafi et al. [46] stated that confidence in novice nurses is an essential factor that affects their clinical performance. Additionally, Makarem et al. [47] noted that professional confidence (PC) can impact all aspects of the clinical performance of healthcare providers, including communication with patients, colleagues, and other healthcare team members, all of which affect the quality of patient care. Therefore, improving confidence in novice nurses is recommended to ensure appropriate care [48]. Effective communication is crucial in nursing and can directly impact patient care and outcomes, according to Leonard et al. [49]. Lastly, maintaining patient perspective is considered the foundation of the nursing care concept [50].

Validity refers to the extent of alignment between the measurement tool and the natural world [51]. A panel of 10 experts was selected to evaluate the instrument’s content validity. The fact that these experts were independent of the research team is considered a strength of the study [52]. The content validity of the PCAS-P scale was confirmed based on the opinions of the expert panel (qualitatively), the content validity ratio (CVR), and the content validity index (CVI). The face validity of the scale was also evaluated using face-to-face interviews and quantitative methods. Using the target group for assessing face validity was necessary because no one was more knowledgeable in this area than novice nurses [53]. Ten novice nurses were asked for their opinions, leading to some items being rewritten partially based on their feedback. Apart from these minor changes, all items were deemed acceptable by participants, indicating the appropriate form and content of the scale. None of the items were eliminated at this stage due to their excellent content and face validity.

The purpose of the CFA was to evaluate the construct validity of the PCAS-P assessment tool. Generally, construct validity refers to the extent to which a multi-item scale reflects the hypothetical dimensions of the construct being measured [54]. The results obtained confirmed the three-factor structure reported in the original version. The model showed a good fit, and all model fit indices were satisfactory, with results consistent with the original instrument [16]. Finally, the study’s findings confirmed the convergent and discriminant validity of PCAS-P among Iranian novice nurses. Therefore, the present research findings indicate that PCAS-P is suitable for future studies among Iranian novice nurses.

The reliability analysis indicates that a scale should continuously reflect the structure it measures [51]. The reliability of the PCAS-P was confirmed by calculating Cronbach’s alpha coefficient (α), McDonald’s omega (ω), Coefficient H, and Average Inter-Item Correlation (AIC) (α = 0.947, ω = 0.948, coefficient *H* = 0.971, and AIC = 0.512), indicating a higher level of reliability compared to the original version of the PCAS scale (α = 0.90). It is worth mentioning that the reliability of the Persian version was also higher in all three dimensions compared to the original tool. Therefore, the values of reliability indicators indicate good internal consistency of the scale and sufficient correlation between the items used. Hence, it can be inferred that the various items that make up the scale evaluate similar ideas or concepts.

As a result, the PCAS-P scale has acceptable validity and reliability. The factors “confidence in the provision of care,” “communication,” and “patient perspective” are essential aspects of care delivery in acute conditions. Thus, the PCAS-P scale is a self-report tool consisting of 17 items and three subscales: confidence in the provision of care (10 items), communication (4 items), and patient perspective (3 items). The items are rated on a 4-point Likert scale. For items 1 to 3 as 1 = strongly disagree, 2 = somewhat agree, 3 = agree, 4 = strongly agree and items 4 to 17 as 1 = very poor, 2 = poor, 3 = good, 4 = very good. Higher scores indicate a greater perception of ability in acute care situations. Hence, further studies using the PCAS scale are necessary to determine whether this tool is sufficient for evaluating interventions to improve novice nurses’ competence in a clinical setting. The PCAS scale has the potential to be used in assessing educational interventions for novice nurses and as a basis for discussing and reflecting on areas where novice nurses need more support and training.

The findings of this study can serve as a reference for examining the psychometric properties, particularly the validity and reliability of the structure. However, the authors acknowledge several limitations in evaluating the psychometric properties of the PCAS-P scale. For conducting CFA, all participants were selected using a convenience sampling method from educational and therapeutic centers affiliated with a university in Iran. This sampling method may weaken the external validity of the results and limit the generalizability of the findings to some extent. Additionally, self-report data may contain potential biases. Furthermore, this study did not include other forms of construct validity testing, such as concurrent validity. Despite these limitations, a strength of this study was conducting confirmatory factor analysis on 236 Iranian novice nurses. This sample size exceeded the required sample size for CFA. Considering the satisfactory fit of the model and good fit indices values, this current research is the first study to evaluate the psychometric properties of this tool in a country other than Sweden. Therefore, it is necessary to conduct further examination of the psychometric properties of the PCAS scale in groups with different languages and cultures.

## Conclusion

The Persian version of PCAS-P is valid, reliable, and has good psychometric properties. Additionally, this tool can assess the perception of care in acute situations among novice nurses due to the brevity of the items and ease of administration. Therefore, we recommend that larger samples and different hospital departments be used in future research to develop the PCAS-P scale among novice nurses in healthcare settings. Consequently, this study can help healthcare system managers and nursing policymakers identify facilitating factors, use its dimensions to ensure the health and safety of high-risk patients, examine strengths and weaknesses, and improve them. Moreover, using the PCAS-P scale provides a suitable opportunity for creating more cross-cultural studies between Iran and other countries. Therefore, using this reliable tool can lead to valuable results regarding the perception of care in acute situations.

### Electronic supplementary material

Below is the link to the electronic supplementary material.

Supplementary Material 1

## Data Availability

The datasets used and analyzed during the current study are available from the corresponding author upon reasonable request.

## Abbreviations

PCAS: Perception to Care in Acute Situations
CVR: Content Validity Ratio
CVI: Content Validity Index
RMSEA: Root Mean Square Error of Approximation
CFA: Confirmatory Factor Analysis
GFI: Goodness of Fit Index
CFI: Comparative Fit Index
TLI: Tucker-Lewis Index
TLI: Tucker-Lewis Index
IFI: Incremental Fit Index
AGFI: Adjusted Goodness of Fit Index
PNFI: Parsimonious Normed Fit Index (PNFI)
ICC: Intraclass Correlation Coefficient
MLE: Maximum Likelihood Estimation
AIC: Average Inter-Item Correlation
CR: Composite Reliability
AVE: Average Variance Extracted
MSV: Maximum Shared Squared Variance
