# Supplementary material for: Translation and validation of the Persian version of the perception to care in acute situations (PCAS-P) scale in novice nurses
Source: BMC Nurs. 2024 Feb 8;23:108. doi: 10.1186/s12912-024-01760-z (PMC10851513; doi:10.1186/s12912-024-01760-z)
Supplement: Supplementary file 1 — Supplementary Material 1 [file 12912_2024_1760_MOESM1_ESM.docx]

Sincerely, the following questionnaire has been designed to carry out a research project entitled "**Translation and Validation of the Persian Version of the Perception to Care in Acute Situations (PCAS-P) Scale in Novice Nurses**". At this phase, the researchers intend to determine the face validity (qualitative and quantitative methods) of the scale. Also, specify how important it is to include the following items in the questionnaire. Of course, having your valuable opinions will help us correct the weak points of the questionnaire implementation and draw better conclusions from this plan. Therefore, please, after reading the questions, give your opinion by choosing one of the options below (using a cross (×) and in the designated places). We sincerely thank you for your cooperation.

**Qualitative phase (face-to-face interview):**

1. Are the components of the measure (e.g., items) relevant to what’s being measured?

2. Does the measurement method seem useful for measuring the variable?

3. Is the measure seemingly appropriate for capturing the variable?

4. In your opinion, how are the items in terms of **ease of response**?

5. In your opinion, how are the items in terms of **relevancy**?

6. In your opinion, how are the items in terms of **ambiguity?**

**Quantitative phase:**

| Face validity | Impact score | | | | |
| --- | --- | --- | --- | --- | --- |
| Items | **Quite important** | **Important** | **almost important** | **a little important** | **not important** |
| 1. I am concerned about providing care in acute situations |  |  |  |  |  |
| 2. I trust my ability to provide care in acute situations |  |  |  |  |  |
| 3. I have sufficient knowledge to provide care in acute situations |  |  |  |  |  |
| 4. I assess my overall ability to provide care in acute situations |  |  |  |  |  |
| 5. I assess my ability to manage the demands I have on myself in acute situations |  |  |  |  |  |
| 6. I assess my ability to manage the demands of my colleagues in acute situations |  |  |  |  |  |
| 7. I assess my ability to independently determine necessary actions in acute situations |  |  |  |  |  |
| 8. I assess my ability to independently prioritize actions in acute situations |  |  |  |  |  |
| 9. I assess my ability to receive instructions over the phone in acute situations |  |  |  |  |  |
| 10. I estimate my ability to carry out instructions that I have received over the phone in acute situations |  |  |  |  |  |
| 11. I estimate my ability to receive instructions from an attending doctor in acute situations |  |  |  |  |  |
| 12. I assess my ability to independently guide clinical care in acute situations |  |  |  |  |  |
| 13. I assess my ability to report patient status to a nurse in acute situations |  |  |  |  |  |
| 14. I assess my ability to take part patients in acute situations |  |  |  |  |  |
| 15. I assess my ability to understand the care needs of each patient in acute situations |  |  |  |  |  |
| 16. I assess my ability to understand the medical needs of each patient in acute situations |  |  |  |  |  |
| 17. I assess my ability to provide information tailored to individual needs in acute situations |  |  |  |  |  |
